# Call combination in African forest elephants *Loxodonta cyclotis*

**DOI:** 10.1371/journal.pone.0299656

**Published:** 2024-03-18

**Authors:** Daniela Hedwig, Anna Kohlberg

**Affiliations:** 1 Elephant Listening Project, K. Lisa Yang Center for Conservation Bioacoustics, Cornell Lab of Ornithology, Cornell University, Ithaca, New York, United States of America; 2 Department of Fisheries, Wildlife, and Conservation Sciences, Oregon State University, Corvallis, Oregon, United States of America; Universita degli Studi di Milano, ITALY

## Abstract

Syntax, the combination of meaning-devoid phonemes into meaningful words, which in turn are combined in structurally and semantically complex sentences, is fundamental to the unlimited expressiveness of human languages. Studying the functions of call combinations in non-human species provides insights into the evolution of such syntactic capabilities. Here, we investigated the combination of high amplitude broadband calls with low frequency rumble vocalizations in a highly social species, the African forest elephant *Loxodonta cyclotis*. Rumbles play an integral role in coordinating social interactions by transmitting socially relevant information, including individual identity. By contrast, broadband calls, such as roars, are thought to function as signals of distress and urgency as they are typically produced in situations of high emotional intensity. Functional changes associated with the combination of these calls remain little understood. We found that call combinations were produced by all age-sex classes but were most prevalent in immature individuals. We found that rumbles used singularly occurred in all five investigated social contexts, whereas single broadband calls were restricted to two resource-related contexts. Call combinations also occurred in all five contexts, suggesting an increase in the functional use of broadband calls when combined with rumbles, analogous to the generativity brought about through syntax in human speech. Moreover, combining calls appeared to lead to functional shifts towards high-stake contexts. Call combinations were more likely in competition contexts compared to single rumbles, and more likely in separation contexts compared to single broadband calls. We suggest that call combination in forest elephants may aide to reduce message ambiguity in high-stake situation by simultaneously communicating distress and individual identity, which may be critical to secure access to resources, reduce the risk of injury and to reunite with or recruit the support of the family group.

## Introduction

The uniquely expressive nature of human language arises from a flexible but rule-governed combination of a finite number of acoustic elements into an unlimited number of semantically and structurally complex expressions [1]. Intriguingly, call combinations are found in a taxonomically wide range of vertebrate species suggesting that the ability to combine acoustic units into larger utterances is evolutionary deeply rooted, yet possible selective driving forces remain little understood. One prevailing hypothesis is that the emergence of call combination and the associated increased expressiveness is driven by social complexity because socially complex societies and contexts may require more nuanced and richer communication systems [2]. For instance, in chimpanzees call combinations appear to function as social tools as call combinations predominantly occur in social vs non-social contexts and are mainly produced by socially challenged individuals, such as females and low ranking males [3]. An alternative, but mutually non-exclusive, hypothesis puts forth communicative efficiency as a driving force behind the evolution of combinatorial calls. Vocal tracts are inherently limited in the number of distinguishable sounds they can produce, and therefore combining existing calls, as opposed to creating new calls, allows for more precise communication and reduces the risk for perceptual errors [4, 5].

Generativity emerges on two syntactic levels in the human language, which is referred to as duality of patterning [6] and defined by the following two principles: 1) meaning-devoid phonemes are combined into meaningful words; and 2) these words are combined into sentences, i.e., compositional messages derived from the meaning of the constituent words. Aiming to discern evolutionary pathways to syntax in human language, pioneering studies of syntax-like structures in non-human vocal communication systems attempted a classification of call combinations analogues to the duality of patterning [6] in human language [7]. The aim was to distinguish whether these call combinations represent analogues to human words or human sentences; the former consisting of acoustic elements that are not used on their own and, as such, are meaning-devoid themselves, the latter representing compositional messages consisting of acoustic elements that can occur singularly and, as such, do carry independent meaning. Comparative research investigating the combinatorial structure and functions of call combination in non-human species has unveiled considerable semantic and structural diversity [reviewed in 8], suggesting various convergent evolutionary processes. Analogies to “words”–meaningless acoustic units arranged in meaningful combination–have thus far been found in a number of bird species [e.g., chickadees: [9, 10], while evidence of various forms of contextual modification associated with the combination of meaningful call types exist in a broad range of species [e.g., gorillas: [11]; meerkats: [12]; chimpanzee: [13]; pied babbler: [14]; Campbell’s monkey: [15]. However, truly compositional call combinations–combining the meaning of singular calls into larger structures that reflect the meaning of their components–appears to be rare, with evidence currently restricted to two bird species and chimpanzees [16–18]. Investigating contextual changes associated with the combination of different call types across species is a fundamental step towards discerning different combinatoriality systems and potential convergent mechanisms in the evolution of syntactic communication systems.

Elephants exhibit one of the most complex social systems among mammal species centered around family groups consisting of related adult females and their offspring [19, 20] with vocal communication playing a critical role in maintaining long-lasting social bonds between females [21–23]. All elephant species (African forest elephants *Loxodonta cyclotis*, African savanna elephants *Loxodonta africana*, and the Asian elephant *Elephas maximus*) frequently combine characteristic tonal low-frequency rumble vocalizations with noisy broadband calls (mainly roars, but also barks and cries) [24–26]. While African savanna and Asian elephants appear to favor particular sequential arrangements (typically a broadband call followed by rumble), African forest elephants appear to produce different sequential arrangements more flexibly and more frequently. This dissimilarity between the two closely related African species suggests that socio-ecological rather than phylogenetic factors drive the evolution of call combinations within the taxon Proboscidea [27]. Various functions have been suggested for elephant call combinations, yet detailed studies are lacking. The use of loud broadband calls, such as roars, and rumbles in elephants appears to be consistent with ‘motivation-structural rules’ underlying the production of mammalian vocalizations [28–30]. Roars have been suggested to signal a caller’s elevated arousal state, distress and urgency as they are predominantly produced in the context of heightened negative arousal, such as aggression, and often accompanied by visual signals of distress [24, 26, 31, 32]. In contrast, rumbles, the most common component of elephant vocal behavior, are associated with a wide range of generally non-aggressive social contexts. They have integral functions in coordinating the social interactions of elephants within and between family groups [23, 33–37]. Rumbles encode the emotional state of the caller [35] and individual identity enabling elephants to recognize each other acoustically over large distances [23]. The few reports on roar-rumble combinations in African savanna and Asian elephants suggest a broad contextual use serving various social functions, and a particular importance for young elephants [24–27, 31].

Here, we investigated acoustic, contextual, and developmental variation associated with the combination of broadband calls and rumbles in African forest elephants. Our goal was to discern the functions of call combination in this highly social species and to investigate potential evolutionary drivers behind the evolution of combinatorial call systems. In accordance with the increased generativity brought about through syntactic systems, we predicted that combining a broadband call with a rumble will increase their functional use. In particular, if call combinations represent compositional messages (i.e. their information content derives from the information content of the constituent call types), we predicted that 1) broadband calls and rumbles used singularly and in combination constitute the same call types and as such will not be structurally different, and 2) the contexts in which combinations are used reflect the contexts of broadband calls and rumbles used singularly. Following the social complexity hypothesis, we predicted that call combinations will be produced predominantly in social contexts and by adult females. Following the communication efficiency hypothesis, we predicted call combinations to be used particularly in contexts in which the reduction of perceptual errors is critical, for instance during competitive encounters or during separation.

## Methods

### Study site

Data collection took place at Dzanga Bai, a forest clearing in the Dzanga-Ndoki National Park in south-western Central African Republic (2.963° N, 16.365° E). The clearing is characterized by a sandy pan of approximately 10 hectares in size intersected by a permanent stream. Dzanga Bai hosts the largest known aggregation of forest elephants with more than 2,000 individual forest elephants visiting each year [38]. Elephants aggregate in the clearing primarily to access mineral-rich water through small monopolizable depressions or pits that the elephants dig themselves [39].

### Data collection

Observations of elephant behavior were conducted by DH from an 8-m high platform located on the edge of the clearing from September 2018 to April 2019, typically from 13:30 and 17:00 hours, when the largest numbers of elephants are visible in the clearing. Data on call context was collected using opportunistic sampling focused on audible calls for which the caller and context were discernable with a high level of confidence. Audio recordings were conducted simultaneously with an Earthworks omnidirectional microphone attached to a Sound Devices MixPre3 Audio recorder at a 48 kHz sampling rate. For each vocalization, ten variables were coded to describe the immediate context of vocal production (Table 1).

**Table 1:** Variables used to describe the contextual use of rumbles, broadband calls and their combinations.

Variable	Description
Sex of caller	Male, female.
Age of caller	Young (incl. infants and juveniles), subadult, adult.
Competition	Conflict over access to resources, mostly mineral pits, occasionally grass. Includes displacements, avoidance interactions, nonphysical and physical aggression, as well as disputes by individuals sharing a mineral pit. Vocalizer typically the displaced individual.
Affiliative	Individuals in the clearing, or upon entering approaching one another, or walking up to a stationary individual, usually leading to benign physical contact. Individuals may stay in proximity, feeding, traveling, resting, or sharing a mineral pit.
Separation	Individuals travelling alone at a large distance from associates or when associates have left the clearing. Individuals often wander around aimlessly, smelling ground with their trunk, frequently stopping to listen, or running in distress with ears and tail erect.
Nursing	Calf approaching or walking parallel along mother’s side with trunk raised, often touching mother’s side, leg or breast. The mother usually stops and starts nursing.
Logistics	Concerted group movement and group coordination as individuals travel or leave the clearing together.
Sexual*	Female presenting her hindquarters allowing males to inspect genitals, often urinating while doing so. Males contesting over females. Females, often in distress, avoiding a male who is pursuing them.
Anti-predator*	Individual mobbing (charging, kicking dust or flinging trunk) animal of other species, or when in a state of alert (ears erect and tail up) upon visitors arriving at the observation platform.

Context descriptions after Hedwig et al. [40]. Age classification after Turkalo [41]: *infant*: ≤ 1 year, still nursing; *juvenile*: ≤ 5–6 years, feeds independently but still nursing; *subadult*: ≤ 10–20 years, independent by 13; *adult*: ≥ 22 years or traveling with dependent calf.

* not included in statistical analyses due to small sample sizes

### Acoustic analysis

Acoustic analysis was conducted on spectrograms generated using a Hann window with a frequency and time resolution of 0.98Hz and 0.0255s in Raven Pro Sound Analysis Software (K. Lisa Yang Center for Conservation Bioacoustics at the Cornell Lab of Ornithology, 2023). Based on visual inspection of spectrograms, vocalizations were classified as single rumbles, single broadband calls and combinations thereof with different sequential arrangement (e.g., B_R represents a combination of a broadband call followed by a rumble). Following Pardo et al. [27], we identified a combination call as any vocal sequence of at least one rumble-like component and at least one roar, bark, or cry-like component in which the constituent parts are immediately adjacent to one another with no temporal overlap and no intervening silence. The onset and offset of rumbles and broadband components were readily discernable through the presence/absence of harmonic structure in the lower frequencies. Similar to Pardo et al. [27], combination type classification was highly reliable with 97% agreement between two observers independently scoring a subsample of 75 calls [40]. A total of 760 calls were used for analysis for which vocalization type was reliably discernable (i.e., no overlapping background noise or other vocalization). In the following we refer to rumbles and broadband calls used singularly and not as part of a combination call as “single rumbles” and “single broadband calls”, rumble and broadband components of combination calls as “combined rumbles” and “combined broadband calls”, and any combination of rumble and broadband call as “combination call” or “RB combination”.

To compare the acoustic structure of single and combined rumbles and broadband calls, we selected a subsample of vocalizations (single rumbles: N = 324; combined rumbles: N = 215; single broadband calls: N = 76; combined broadband calls: N = 158). We measured six acoustic parameters describing the duration and energy distribution of vocalizations (Table 2). Acoustic measurements were based on measurement selection boxes drawn in Raven Pro extending along the temporal axis from the start to the end of the single or combined call while its upper and lower boundaries enclosed a frequency range from 10Hz to 250Hz for rumbles and 10Hz to 1000Hz for broadband calls.

**Table 2:** Acoustic parameters measured for single and combined forest elephant rumbles and broadband calls.

Parameter	Definition
Call duration	Duration of call containing 90% of energy, calculated as the difference between the time of the location of the 95% and 5% percentile of energy within the measurement box
Peak frequency	Frequency with highest energy
Location of peak frequency	Position of frequency with highest energy, as percentage of call duration
Location of 25% of energy	Position within call at which 25% of energy is reached, as percentage of call duration
Location of 50% of energy	Position within call at which 50% of energy is reached, as percentage of call duration
Location of 75% of energy	Position within call at which 75% of energy is reached, as percentage of call duration

To account for correlations between the six acoustic parameters we performed Principal Components Analyses (PCA) using the R package *psych* [42]. We performed PCA first with all six parameters includes, examined the Eigenvalues and the loadings of the parameters, then reduced the number of parameters with the goal to maximize the variance explained by the Principal Components [43]. Results from the PCA for the six parameters measured for single and combined rumbles suggested combining the four parameters describing the distribution of energy within the call (loadings > 0.5; location of peak frequency, location of 25% of energy, location of 50% of energy, location of 75% of energy) into one variable “Energy distribution”. Similarly, PCA for the six parameters measured for single and combined broadband calls suggested combining two parameters describing the distribution of energy early within the call (loadings > 0.64; location of 25% of energy, location of 50% of energy) into one variable “Energy distribution”.

We used two approaches to examine whether certain acoustic parameters discriminate between single and combined rumbles, and single and combined broadband calls. First, we used Mann-Whitney U tests with the function “wilcox.test”, in conjunction with a Bonferroni Correction to account for multiple tests [44]. All Bonferroni corrections were conducted by multiplying the obtained p-values by the number of tests, while maintaining a significance threshold of 0.05. Adjusted p-values resulting in values >1 were truncated to “1” [45]. Second, we performed linear discriminant function analysis with the function “lda” of the R package MASS [46] to investigate how accurately rumbles and broadband calls can be classified as “single” versus “combined”, and evaluated the classification accuracy with a leave-one-out cross validation method (jack knife reclassification [47]. Because unbalanced datasets, as in our case, lead to skewed prior classification probabilities [48], we determined whether the classification accuracy was significantly higher than expected based only on the prior classification probabilities.

### Context differences

To investigate contextual use, we pooled all sequential arrangement of broadband calls and rumbles into one category “RB combinations”. We compared the contextual use of RB combinations, single broadband calls, and single rumbles based on a subset of calls for which we were able to identify the context (N = 727 calls). Call contexts with fewer than ten observations were excluded from analysis (unspecific: five single rumbles; sexual: eight single rumbles and one RB combination; antipredator: three single rumbles), resulting in a dataset of N = 710. We used Fisher’s Exact test, implemented in the function fisher.test in the package “nnet” [46] and the function fisher.multcomp in the “RVAideMemoire” package [49] to perform post-hoc pairwise comparisons of proportions with a Bonferroni correction. We chose Fisher’s Exact test because complete separation (i.e., some call types were exclusively observed in one of the contexts) prevented us from deriving meaningful estimates using a regression analysis.

### Age-sex differences

To investigate age-sex-specific differences in the use of RB combinations, single broadband calls and single rumbles we selected a subset of calls for which the callers’ age-sex class was known (N = 652). We used multinomial logistic regression [50] implemented in the function “multinom” provided by the “nnet package” [46]. Call type was specified as the categorical response variable (three levels: RB combination, single broadband call, single rumble) and the age-sex class of the caller as the categorical predictor variable (six levels: adult females, subadult females, young females, adult males, subadult males, young males). As an overall test of the significance of the predictor, we compared the model with the age-sex predictor to a null model without a predictor variable using a likelihood ratio test [51] and the R function “anova”. We calculated p-values for the regression coefficients using Wald tests with a 0.05 significance level.

The statistical tests used for our analyses were performed in software environment R, version 4.0.3 (R Core Team, 2020).

### Ethical note

This work was carried out using a non-invasive observational method, which required no direct contact with the animals, and in accordance with the national laws of the Central African Republic. Research clearance was approved by the Central African government’s Ministry for Water, Forest, Hunting and Fishing as well as the Ministry for Education and Scientific research.

### Inclusivity in global research

Additional information regarding the ethical, cultural, and scientific considerations specific to inclusivity in global research is included in the Supporting Information (S1 Checklist).

## Results

Single rumbles were by far the most frequently observed call type, followed by RB combinations. Five types of RB combinations were observed with varying frequency, with broadband calls followed by a rumble (“B_R”) being the most common (N = 760; Table 3).

**Table 3:** Frequency of observations of rumbles (“R”), broadband calls (“B”) and RB combinations with different sequential arrangement listed separately below.

Call type	# Observations
R	398
B	135
RB combinations	227
B_R	110
R_B_R	66
R_B	47
B_R_B	3
R_B_R_B	1

### Acoustic structure

**Fig 1:**
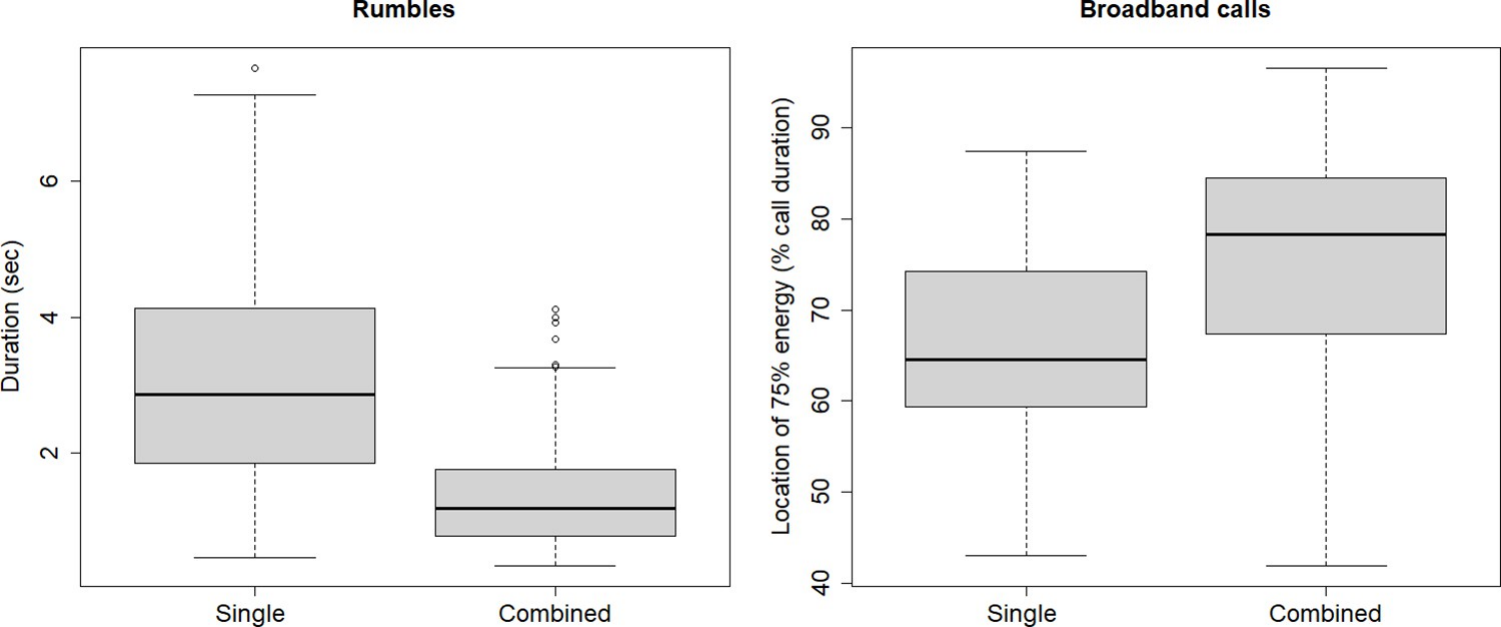
Differences in the acoustic structure of single rumbles as compared to combined rumbles, as well as between single and combined broadband calls.

We found significant differences in the acoustic structure of single rumbles as compared to combined rumbles, as well as between single and combined broadband calls. Combined rumbles were significantly shorter in duration and higher in peak frequency compared to singles rumbles but did not differ in their temporal energy distribution (Table 4; Fig 1; Mann-Whitney U-test with Bonferroni Correction for three comparisons; duration: W = 58774, p> 0.001; peak frequency: W = 25770, p> 0.001; energy distribution: W = 34263, p> 1). Discriminant Function Analysis was significantly better than chance in assigning rumbles as single or combined (p< 0.001), with a classification accuracy of 78% (single rumbles: 81% (prior probability: 60%); combined rumbles: 75% (prior probability: 40%)). Duration carried the most weight in the discrimination (coefficients of linear discriminant function: duration: -0.72; peak frequency: 0.01; energy distribution: 0.04).

**Table 4:** Acoustic characteristics of single and combined rumbles and broadband calls measured based on six acoustic parameters.

	Rumbles		Broadband calls	
Parameter	Single	Combined	Single	Combined
**Call duration**	3.06	1.38	1.65	1.46
	(0.46–7.67)	(0.34–4.12)	(0.46–4.91)	(0.2–3.18)
**Peak frequency**	70.58	110.55	578.14	548.04
	(10.74–250)	(10.74–250)	(223.63–993.16)	(219.73–1000)
**Location of peak frequency**	43.64	49.1	51.74	57.98
	(0.45–99.75)	(0.12–99.54)	(14.4–84.27)	(0.53–99.71)
**Location of 25% of energy**	31.08	30.34	37.27	36.83
	(6.56–72.21)	(3.33–83.34)	(17.22–68.43)	(12.65–72.99)
**Location of 50% of energy**	47.49	47.54	51.9	57.29
	(14.18–80.04)	(6.79–94.43)	(27.91–78.32)	(26.38–92.5)
**Location of 75% of energy**	64.93	65.61	66.31	76.04
	(29.42–92.69)	(26.31–99.54)	(43.08–87.45)	(41.91–96.57)

Single and combined broadband calls showed significant differences in their temporal energy distribution, but not in other measured acoustic parameters. Combined broadband calls showed an even distribution of energy across the call with 75% of energy being reached on average after 75% of call duration. Energy in single broadband calls was shifted towards call onset with 75% of energy already being reached on average at 66% of call duration (Table 4; Fig 1; Mann-Whitney U-test with Bonferroni Correction for five comparisons; duration: W = 6656, p = 0.9; peak frequency: W = 6490.5, p> 1; location of peak frequency: W = 5077.5; p = 0.3; energy distribution: W = 5284, p = 0.7; location of 75% of energy: W = 3105.5, p> 0.001). Discriminant Function Analysis was significantly better than chance in assigning broadband calls as single or combined (p< 0.001), with a classification accuracy of 76% (single roars: 50% (prior probability: 32%), combined roars: 88% (prior probability: 68%)). Energy distribution and duration carried particular weight in the discrimination (coefficients of linear discriminant function: energy distribution: -0.94; duration: -0.73; location of 75% of energy: 0.14; location of peak frequency: -0.006; peak frequency: < 0.001).

### Context differences

**Fig 2:**
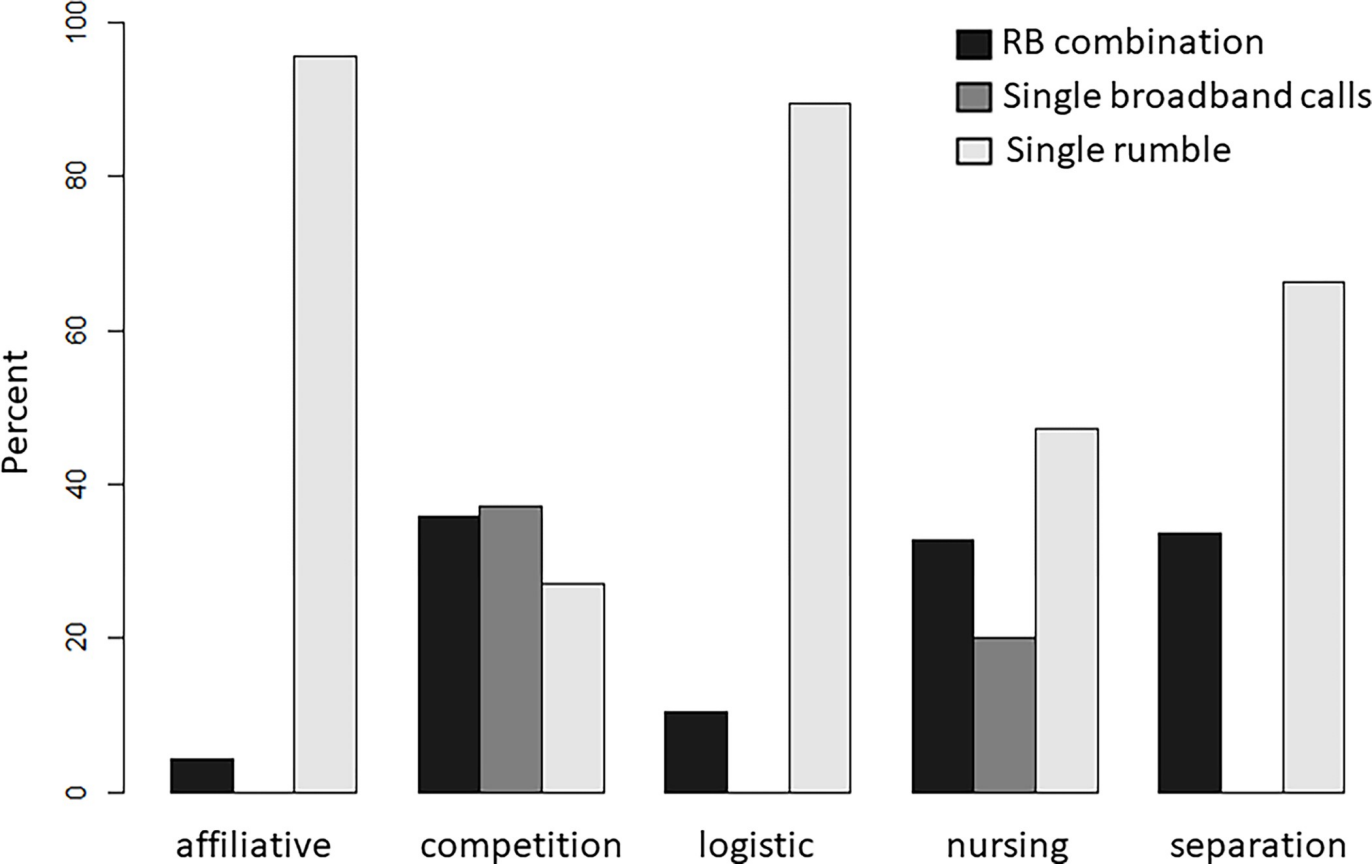
Contextual use of RB combinations, single broadband calls, and single rumbles produced by forest elephants.

RB combinations, single broadband calls, and single rumbles showed significant differences in their contextual use (Fisher’s exact test; p = 0.0002, Table 5, Fig 2). The pairwise comparisons between the five context categories and the three call types revealed only two significant differences between the contextual use of single roars and RB combinations, but five significant differences between single roars and single rumbles as well as RB combinations and single rumbles (Table 5).

**Table 5:** Results of pairwise comparisons using fisher’s exact tests to investigate if the proportion of calls belonging to three call types differed between five context categories. P-values were corrected for multiple testing using a Bonferroni correction for 30 pairwise comparisons. Adjusted p-values resulting in values >1 were truncated to 1.

	RB combination vs. single rumble	RB combination vs. single broadband call	Single broadband call vs. single rumble
Affiliative vs. competition	**<0.001**	1	**<0.001**
vs. logistics	1	-*	-*
vs. nursing	**<0.001**	1	**<0.001**
vs. separation	**<0.001**	-*	-*
Competition vs. logistics	**0.004**	1	**<0.001**
vs. nursing	1	1	0.063
vs. separation	**<0.001**	**<0.001**	**<0.001**
Logistic vs. nursing	0.597	1	0.332
vs. separation	1	-*	-*
Nursing vs. separation	1	**<0.001**	**<0.001**

* single broadband calls were observed in neither context

#### Single broadband calls vs. single rumbles

While single rumbles were observed in all five contexts, broadband calls were restricted to competitive interactions and the nursing context. The proportion of single broadband calls and single rumbles significantly differed between competition contexts and all other contexts but nursing: single broadband calls were more likely in competition contexts compared to single rumbles, whereas rumbles were predominantly used in logistic, separation and affiliative contexts. We also found significant differences between the nursing context and the separation and affiliation contexts. Broadband calls did not occur in separation and affiliation contexts but were half as likely as single rumbles in nursing contexts (ratio of single broadband calls to single rumbles; competition: 1.38; nursing: 0.42; Table 5, Fig 2).

#### RB combinations vs. single rumbles

As with single rumbles, RB combinations occurred in all five contexts. Their proportions showed significant differences between the competitive context and all other contexts. Similar to single broadband calls, RB combinations were more likely than single rumbles in competition contexts, while single rumbles were more likely in all other contexts. We also found significant differences in the proportion of RB combinations and single rumbles between affiliative contexts and nursing as well as separation contexts. While single rumbles were 22 times more likely than RB combinations in affiliative contexts, they were approximately only twice as likely during nursing and in separation contexts (ratio of RB combinations to single rumbles; separation: 0.51; affiliative: 0.05; competition: 1.33; logistic: 0.12; nursing: 0.69; Table 5; Fig 2).

#### RB combinations vs. single broadband calls

The proportion of RB combinations and single broadband calls differed significantly between the nursing and competition contexts and the separation context, in which RB combinations were more likely (ratio of RB combinations to single broadband calls; competition: 0.97; nursing: 1.64; Table 5; Fig 2).

### Age-sex differences

**Fig 3:**
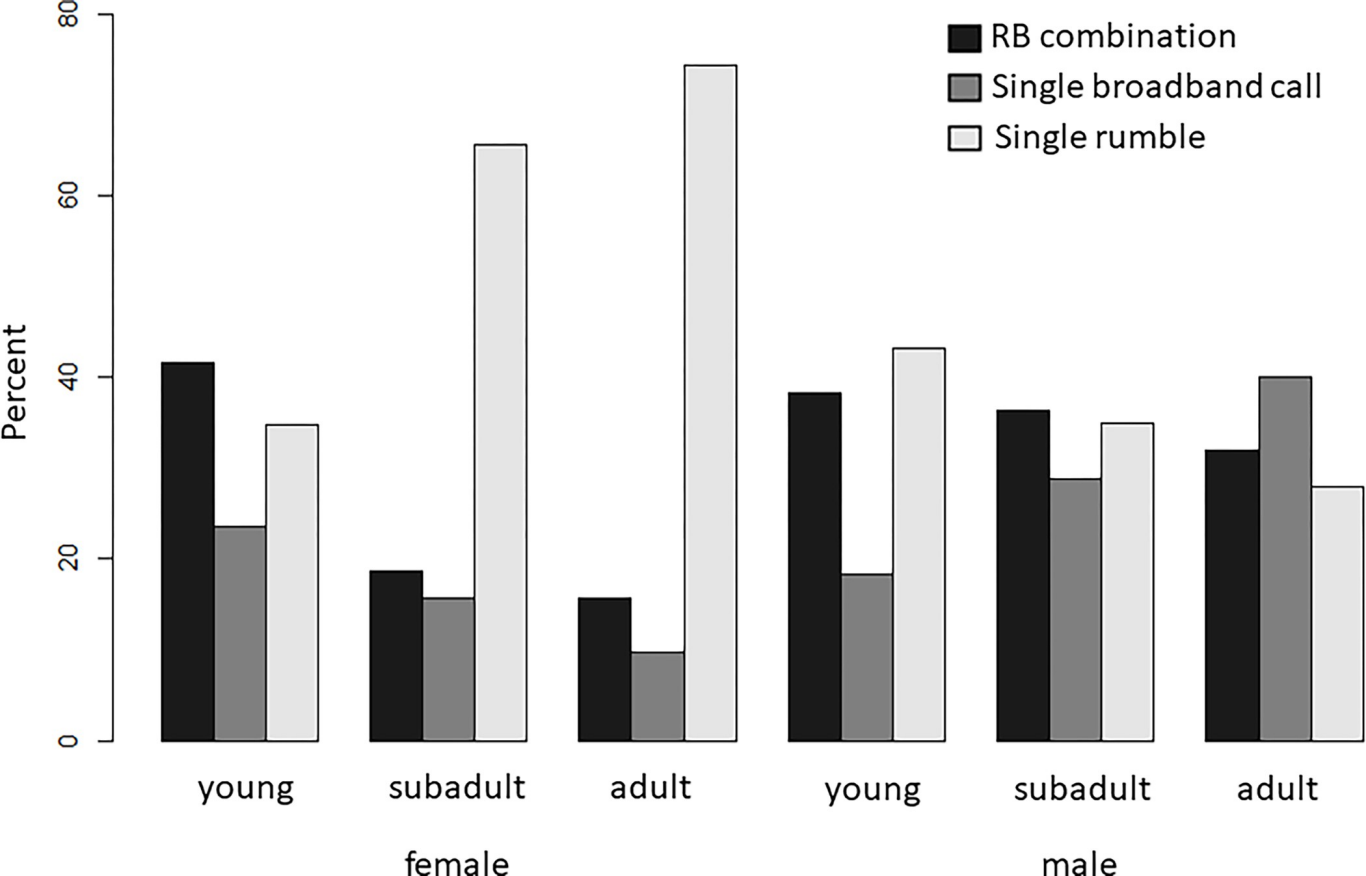
Age-sex differences in the use of RB combinations, single broadband calls, and single rumbles in African forest elephants.

RB combinations, single broadband calls, and single rumbles were produced by all male and female forest elephants of all age classes, but our analyses revealed significant differences in the frequencies with which these call types were observed across age and sex groups (likelihood ratio test full vs. null model: *Χ*^2^ = 80.781, p< 0.001; Table 6, Fig 3).

**Table 6:** Results of the multinomial logistic regression investigating differences between age-sex groups in the use of single rumbles, single broadband calls and RB combinations.

	Single broadband calls vs single rumbles					RB combinations vs single rumbles					RB combination vs single broadband calls			
	Estimate	SE		P-value		Estimate		SE	P-value		Estimate	SE		P-value
**Adult female vs**														
(Intercept)	-2.030	0.295		0.000		-1.551		0.240	0.000		0.480	0.353		0.174
Young females	**1.641**	**0.409**		**0.000**		**1.728**		**0.342**	**0.000**		0.087	0.446		0.846
Subadult females	0.597	0.382		0.118		0.292		0.330	0.376		-0.305	0.461		0.508
Young males	**1.177**	**0.386**		**0.002**		**1.433**		**0.312**	**0.000**		0.256	0.435		0.556
Subadult males	**1.836**	**0.361**		**0.000**		**1.589**		**0.310**	**0.000**		-0.247	0.409		0.546
Adult males	**2.387**	**0.574**		**0.000**		**1.684**		**0.571**	**0.003**		-0.703	0.591		0.235
**Adult male vs**														
(Intercept)	0.357	0.493		0.469		0.134		0.518	0.796		-0.223	0.474		0.638
Young females	-0.746	0.568		0.189		0.043		0.572	0.940		0.789	0.547		0.149
Subadult females	**-1.790**	**0.549**		**0.001**		**-1.392**		**0.565**	**0.014**		0.397	0.559		0.477
Young males	**-1.210**	**0.552**		**0.028**		-0.252		0.554	0.650		0.959	0.538		0.075
Subadult males	-0.551	0.535		0.303		-0.095		0.553	0.863		0.456	0.517		0.379
**Young females vs**														
(Intercept)	-0.389	0.283		0.168		0.177		0.243	0.467		0.566	0.273		0.038
Subadult female	**-1.043**	**0.373**		**0.005**		**-1.435**		**0.333**	**0.000**		-0.392	0.403		0.330
Young males	-0.464	0.377		0.218		-0.295		0.314	0.348		0.169	0.373		0.650
Subadult males	0.195	0.351		0.578		-0.138		0.313	0.658		-0.334	0.343		0.330
**Subadult females vs**														
(Intercept)	-1.433	0.243		0.000		-1.258		0.227	0.000		0.174	0.296		0.556
Young males	0.579	0.348		0.096		**1.141**		**0.301**	**0.000**		0.561	0.390		0.150
Subadult males	**1.239**	**0.320**		**0.000**		**1.297**		**0.300**	**0.000**		0.058	0.361		0.872
**Young males vs**														
(Intercept)	-0.853	0.249		0.001		-0.118		0.198	0.553		0.736	0.254		0.004
Subadult males	**0.659**	**0.325**		**0.042**		0.156		0.279	0.575		-0.503	0.327		0.124

#### Single broadband calls vs. single rumbles

Adult and subadult females showed significant differences in their proportion of single broadband calls and single rumbles compared to all other age-sex groups. We also found significant differences in the proportion of single broadband calls and single rumbles in subadult males compared to young females and young males, as well as adult males compared to young males. Single broadband calls were four and seven times less likely than single rumbles in subadult and adult females, respectively, but 1.5 times more likely than single rumbles in adult males. Single broadband calls were generally less likely than single rumbles in all other non-adult age-sex classes, yet more likely than in adult and subadult females. Single broadband calls were significantly more likely compared to single rumbles in adult males compared to young males, but significantly less likely in young males compared to subadult males (ratio of single broadband calls versus single rumble; adult female: 0.13; subadult female: 0.24; young female: 0.68; adult male: 1.43; subadult male: 0.82; young male: 0.43; Fig 3, Table 6).

#### RB combinations vs single rumbles

Similar to single broadband calls, adult and subadult females showed significant differences in their proportion of RB combinations and single rumbles compared to all other age-sex groups. While RB combinations were three and four times less likely than single rumbles in adult and subadult females, RB combinations were either approximately equally or more likely than single rumbles in other age-sex groups (ratio of rumble-roar combinations versus single rumbles; adult female: 0.21; subadult female: 0.28; young female: 1.19; adult male: 1.14; subadult male: 1.04; young male: 0.89; Fig 3, Table 4).

#### RB combinations vs single broadband calls

We found no significant differences in the proportion of RB combinations and single broadband calls between any of the age-sex groups. All age-sex groups (apart from adult males) exhibited a larger proportion of RB combinations as opposed to single roars (ratio of rumble-roar combinations versus single roars; adult female: 1.62; subadult female: 1.92; young female: 2.85; adult male: 0.62; subadult male: 4.08; young male: 3.69; Fig 3, Table 4).

## Discussion

A fundamental component of human language is to generate meaningful expressions through the combination of acoustic elements. The study of call combinations in non-human animal species provides insight into not only the functions of a species’ vocal communication system, but also the selective drivers behind the evolution of such syntactic capabilities. Here we investigated the acoustic, contextual, and developmental variation associated with the combination of broadband distress calls and rumble vocalizations in African forest elephants. Forest elephants used single rumbles and broadband calls in starkly different contexts. Single rumbles occurred in all five investigated contexts and were the predominant call type during interactions in affiliative and logistic contexts. Single broadband calls were restricted to two resource-related contexts: competition over access to mineral pits, typically when receiving aggression and/or being displaced; and during nursing, typically when young elephants attempted to gain access to their mother’s breasts. Combination calls occurred in all five investigated contexts, similar to single rumbles. Compared to single rumbles, combinations were overall more likely in competition contexts, and compared to single broadband calls more likely in separation contexts.

What do these findings reveal regarding the functioning of call combinations in African forest elephants? If the described call combinations are truly compositional messages, in that their informational content can be derived from the informational content of their constituent parts, we expected contextual overlap between rumbles and broadband calls used singularly and their combination. As expected for syntactic systems, the combination with a rumble appeared to increase the functional use of the roar, analogous to the generativity brought about through syntax in human speech. In line with compositionality, call combinations occurred in all contexts in which single rumbles and single broadband calls were observed. However, we found significant differences in the acoustic structure between single and combined rumbles, as well as between single and combined broadband calls. These results suggest that broadband calls and rumbles found as part of call combinations may constitute distinct acoustic elements, which only occur in combination and do not carry independent meaning. This controverts the argument that these combinations represent true compositional messages. Instead, the call combinations observed in forest elephants would resemble what Engesser and Townsend [8] described as *multi-element calls*. For example, chestnut-crowned babblers combine meaningless elements to generate meaningful combination calls whereby the sequential arrangement of elements is associated with different functions [52]. Linguistically, these combinatorial systems may be analogous to phonology in human language, where meaningless units (“phonemes”), never used singularly, are combined into meaningful words. On the other hand, the measured parameters showed considerable overlap between combined and single broadband calls and rumbles. Single rumbles are a highly graded group of vocalizations showing continuous acoustic variation across contexts and age-sex classes [53]. Similarly, various kinds of broadband calls have been described [25]. Therefore, it is possible that the rumbles and broadband calls occurring in combination represent subtypes or context-specific variants of rumbles or broadband calls produced singularly. If so, forest elephant call combinations may resemble *intermediate structures*, as described by Engesser and Townsend [8]. For instance, gorillas combine highly intergraded acoustic units, the continuous acoustic variation of which reflects continuous variation in emotional and behavioral state [11]. The resulting combinations show contextual overlap and differences compared to the acoustic units used singularly, which may reflect intermediate emotional states experienced by the caller. Our findings provide first insights into the functioning of call combinations in forest elephants and a critical starting point for future in-depth studies. Detailed studies are needed to compare the structure of combined and single rumbles and broadband calls in particular contexts. Moreover, we investigated combinations broadly without considering the sequential arrangement of rumbles and broadband calls in combination. Further analysis of contextual variation associated with different sequential arrangements in conjunction with playback experiments will shed further light on how call combination generates meaningful messages in forest elephants.

In addition, investigating the contextual use of call combinations in forest elephants allowed us to test two non-mutually exclusive hypotheses regarding potential driving forces behind the evolution of combinatorial call systems. According to the social complexity hypothesis we predicted call combinations to be particularly prevalent in social contexts and predominately produced by adult female forest elephants. Following the communicative efficiency hypothesis, we expected call combinations to occur in high-stake contexts, when precise messaging is critical. Our results provide limited support for the social complexity hypothesis. Both single and combination calls were produced in inherently social contexts, involving direct social interactions. The functional shift of broad band calls to a broad range of social contexts when in combination may indicate a relevance of combinatoriality in navigating complex social interactions, yet, call combinations were not predominately used by adult females, which form the most complex social bonds in the elephant social system. However, the observed contextual shifts of call combinations towards competition and separation contexts compared to call types used singularly support the hypothesis that call combinations may function to reduce ambiguity in the communicated message. These contexts represent situations in which callers experience an elevated risk of injury or losing contact with their family groups. Precise messaging may be critical to secure access to resources, reduce the risk of injury when being displaced from a mineral pit, and to reunite with the family group. Combinatoriality can increase message precision in different ways. For instance, call combinations in forest elephants may combine the information contents of the component calls in an additive fashion, similar to pant hoot and grunt combinations in chimpanzees [13] and close call combinations in Diana monkeys [54]. When receiving aggression during a competitive encounter, adding a roar to communicate distress may more likely elicit the support of kin or remission from further aggression. In turn, when separated, transmitting information on individual identity encoded in a rumble to facilitate recognition by kin is imperative to effectively reunite with family; using a single broadband call to communicate distress alone may be futile. However, due to the attenuation of sound over distance call combinations could serve this function only at a limited spatial range in the forest elephant’s dense rainforest habitat [55]. Future studies need to investigate whether individuals producing combinations in competition, separation, and nursing contexts are indeed less likely to receive physical aggression, more successful at recruiting support and/or gaining access to the mother’s breasts, or able to reunite faster with group members as compared to those using single broadband calls or single rumbles.

Our findings highlight the importance of call combination in forest elephants in coordinating interactions in various social contexts and across all age-sex classes. Call combinations occurred in all investigated contexts and males and females of all age classes used call combinations. All age-sex classes, but adult males, were more likely to use broadband calls in combination with a rumble than as single calls. However, our results indicate sex-specific shifts in the use of single broadband calls and call combinations, as opposed to single rumbles, as elephants mature. Call combinations appeared particularly prevalent in non-adult individuals. In females, the use of combinations and single broadband calls appeared to decrease with age, replaced by the predominant use of single rumbles in adult females. In contrast, as males mature, call combinations and single broadband calls appear to become more prevalent over single rumbles. These sex differences likely reflect the divergent life histories and social behavior of male and female elephants. Males leave their natal groups to become largely solitary, whereas female forest elephants remain with their mothers until they give birth and form their own core families while maintaining strong bonds throughout their lives [38]. Interestingly, our results demonstrate that this shift to adult vocal behavior in females, dominated by the use of rumbles, starts well before the age of primiparity.

Along with an increasing number of similar studies demonstrating call combination in a taxonomically wide range of vertebrate species, our findings in the African forest elephant further underline call combination as an evolutionary deeply rooted communication principle. The enormous structural and semantic variation in call combination systems found across species starkly contrasts the traditional attempted classification of animal call combinations into forms of phonological and lexical syntax analogous to words and sentences in human language [7]. More nuanced categorizations like those attempted by Engesser and Townsend [8] will facilitate broader cross-species meta-analyses investigating the occurrence of different structural and semantic aspects of call combination in relation to the various social and environmental factors that potentially drive the evolution of generative capacity across communication systems.

## Supporting information

S1 Checklist (DOCX)
